# Gendered relations? Associations between Swedish parents, siblings, and adolescents' time spent sedentary and physically active

**DOI:** 10.3389/fspor.2024.1236848

**Published:** 2024-02-22

**Authors:** Sara Hoy, Håkan Larsson, Karin Kjellenberg, Gisela Nyberg, Örjan Ekblom, Björg Helgadóttir

**Affiliations:** ^1^ Department of Movement, Culture and Society, The Swedish School of Sport and Health Sciences (GIH), Stockholm, Sweden; ^2^ Department of Teacher Education and Outdoor Studies, The Norwegian School of Sport Sciences (NIH), Oslo, Norway; ^3^ Department of Physical Activity and Health, The Swedish School of Sport and Health Sciences (GIH), Stockholm, Sweden; ^4^ Department of Global Public Health, Karolinska Institute, Stockholm, Sweden; ^5^ Department of Clinical Neuroscience, Karolinska Institute, Stockholm, Sweden

**Keywords:** physical activity, gender norms, adolescence, motherhood, fatherhood, family

## Abstract

**Introduction:**

The family is assumed to be fundamental in youth socialization processes and development, connected to social and cultural practices such as healthy lifestyles and physical activity. However, gender patterns in physical activity among adolescents and the structural drivers of gender inequality (e.g., parentage and siblingship) are poorly understood. The aim of this study was to explore further how gender structures relate to adolescents' time spent being sedentary and physically active, using contemporary gender theory.

**Methods:**

This cross-sectional study involved 1,139 adolescents aged 13-14 and their parents, including 815 mothers and 572 fathers. Physical activity and time spent sedentary were assessed through accelerometry among adolescents and through a self-report questionnaire for parents validated against accelerometry.

**Results:**

The results showed significant relationships between mothers' moderate-to-vigorous physical activity (MVPA) and girls' MVPA on weekdays and weekends, and fathers' MVPA was significantly related to girls' MVPA on weekdays. Our results imply that the relationship between Swedish parents' and adolescent girls' physical activity in higher intensities are to some extent gendered practices. However, time spent sedentary does not seem to show any patterns of being performed according to binary ideas of gender. Further, our exploratory analyses suggest that these results somewhat intersect with parents' educational level and relate to intra-categorical aspects of doing gender. The results also indicate slight gendered patterns in the “doing” of brotherhood for time spent sedentary, however, for boys only on weekends.

**Discussion:**

The study contributes to the understanding of gender norms as constraints and enablers for adolescents' participation in physical activity. The results can spur public health and physical activity research to apply a contemporary gender theory approach, and to expand the research agenda connected to what relates to gender inequalities in physical activity practices.

## 1 Introduction

Within a Swedish context, a decline in physical activity occurs during adolescence, where girls are less physically active than boys (1). This is mirrored in global research. Importantly, this research also displays that the gap between the two gender categories is increasing, due to that boys have increased their physical activity levels in the last two decades in this age group (2). Recent research reports not only differences between girls and boys, it also displays the differences within groups showing higher variability among boy's moderate-to-vigorous physical activity (3). These results reveal greater variation between individuals among boys, whereas girls showed less variation between them and with a narrower spread of girls centered around median volumes of physical activity (3). This points toward deepened gender inequalities during adolescence, and access to the potential health-promotive benefits of physical activities (4). An influencing factor here is the impact of social norms, where there are social logics of practice connected to gender that contribute to the various ways we participate in physical activities (5, 6). Earlier research reports that girls are perceived as not feminine when practicing certain physical activities, and that being physically active as a girl is challenging gender stereotypes (7). For example, a commonly reported barrier to higher intensities of physical activity among girls is the issue of sweating and being flushed (6). Conversely, boys who are not physically active or do sports are often perceived as not “masculine enough” (8).

As highlighted by Guthold and colleagues (9), gender patterns in physical activity among adolescents are poorly understood, along with the structural “drivers” of gender inequality (e.g., parentage and siblingship). Despite a rather long rationale for further investigating the issue (9), contemplating the use of gender-theory-informed research is not brought up, even though it is an area of research that has a long tradition of studying gender patterns from a relational perspective. Numerous public health researchers have critiqued public health research's lack of theoretical clarity (10) and absence of contemporary gender-theory despite that international research shows substantial gendered patterns within several health topics (11). As an example, only one out of the twelve studies included in Martin and colleagues' systematic review on adolescents' perspectives on barriers and facilitators for physical activity used a gender-theory approach (7). This issue is further accentuated in Miani and colleagues (12) review of how gender is quantitatively assessed as a social factor in health epidemiology, where the majority of 344 measures reviewed lacked theoretical foundation, reinforcing the binary understanding of gender through stereotypes of femininity and masculinity. A joint core in this critique is that the common use of essentialist categorical thinking implies that women and men are viewed as fixed, unproblematic categories, i.e., that gender is something that you are (13). Through exploring the co-occurrences of physical activity (volume and intensity) among adolescents and their parents and siblings, we intend to address this critique in two ways. First, through looking at statistical differences between gender categories with a relational theoretical lens, and second, by exploring intra-categorical aspects of our data. With this approach, we provide an alternative (critical) perspective and give examples of other possible ways of engaging with categorical data.

## 2 Background

The family is assumed to be fundamental in youth socialization processes and development. These processes include the production and reproduction of social and cultural practices such as healthy lifestyles and physical activity (14). Within the family context, parents and siblings are suggested to be connected to how young people establish their relationship with physical activity and sedentary behaviors (15, 16). In a Swedish context, girls coming from family cultures with high socioeconomic status have been shown to be more physically active than girls with low socioeconomic status, while this difference was not found among boys (1). This highlights the relational aspect of how higher intensities of physical activity seem to be performed differently in relationship to family socioeconomic status, however only among girls. Earlier research on facilitating and restraining factors for adolescents' physical activity engagement also shows that the family can be experienced in both a positive and negative way (7). However, knowledge concerning the relationship between the family and adolescents' physical and sedentary behavior, where different family members and gender structures are assessed, is something that would further expand the understanding of young people's physically active lives (17).

Systematic reviews report moderate to strong associations between parental physical activity relate to their young children's activity (18). However, an overall positive yet weak association between parent and adolescent physical activity levels are shown in earlier research (19). The strength of associations between parents' and their children's physical activity seem to change over time in relation to age and is further highlighted in other studies (20). Although parent-child dyad results concerning physical activity and sedentary behavior during adolescence seem somewhat conflicting, the relationship to sociocultural context is rarely addressed (18–20). Additionally, very few studies exploring the adolescent age-group report associations between several “gender combinations” (e.g., mother-daughter, mother-son, father-daughter, father-son) (19). Several other studies have investigated different gender combinations among parents’ and siblings' physical and sedentary behaviors, with varying results and quality (21–24). When Yang and colleagues (25) investigated the association of parents' physical activity trajectories with their children's activity among Finns, they found that the fathers' activity had the strongest association for their daughters' and sons' activity levels, and that mothers' high to moderate activity levels increased the likelihood of their daughters being physically active during youth. Additionally, changes in parental leisure time physical activity were unrelated to changes in their children's youth and adult activity for either gender over time (26). In their trajectory analysis review, Lounassalo and colleagues (27) showed that, e.g., socioeconomic status, being Caucasian, having parent support, and being categorized as male was associated with persistent or increased levels of physical activity across the life-course. This further shows that a physically active lifestyle does not develop uniformly between individuals. A systematic review exploring the topic of siblings and physical activity practices underscores how underdeveloped this area of research is compared to parent-child focused research and adds that there is a need for future theory-driven research in this area (28). However, there is earlier research showing evidence for associations between sibling status and attributes among children and adolescents and their physical activity as well as sedentary behaviors.

### 2.1 Theoretical framework

In this study we will make use of the work by West and Zimmerman (29, 30) and their theorization of *doing gender*, where gender, rather than an essence (something that people “are”), is viewed as a relational category that people perform (or “do”) in and through their actions. Certainly, it is individuals who “do” gender, nevertheless, the doing is socially situated and carried out in presence of others who are presumed to be concerned with its (re)production. The “doing” of gender is viewed as a social practice, an interaction organized in various and manifold activities to reflect or express gender. West and Zimmerman (29) distinguish between the concepts of *sex*, *sex category*, and *gender*; where sex is the socially agreed upon biological criteria for classifying individuals as males or females, while *sex category* presumes an individual's *sex* based on socially required identification. For example, it is possible to claim membership in a *sex category* without exhibiting any *sex* criteria being met. In contrast, *gender* “is the activity of managing situated conduct in light of normative conceptions of attitudes and activities appropriate for one's sex category. Gender activities emerge from and bolster claims to membership in a sex category” (29)*.* In this way, “doing gender” is a continuous activity embedded in everyday interaction, where these normative conceptions vary within sociocultural contexts and over time, i.e., the doing of gender holds spatial and temporal features. This theorization questions the idea of essential criteria, instead, sex, sex category, and gender are seen as socially and culturally constructed events. Actions and activities that individuals do, e.g., being physically active in various ways, are subject to comment and held accountable to interactional and institutionally approved standards (29, 30). From this point of view, a *doing gender* perspective is embedded in a non-essentialist perspective, which emphasizes the relational aspects of how gender is performed in relation to something else within a given space and time, and opposes the idea of an essentialist categorical thinking view of gender (13).

As illustrated by Rhodes and colleagues (16), social norms and culture are societal/macro factors influencing the relationships between family and physical activity and sedentary behavior in youth. This way, sex categories and gender are culturally performed and institutionalized events. Fenstermaker and West (31) refer to this institutional system, and how it assumes particular family structures and a model in which fathers are males and exhibit masculine traits and mothers are females exhibiting feminine traits.

Even if gender-theory is not yet commonly utilized within public health research, West & Zimmerman's theoretical framework is one of the most used ones (11). In general, gender theories are more commonly used in physical education research (e.g., 32). This study makes use of this relational theory in mainly two out of the eight ways mentioned by Hammarström and Hensing (11); to integrate the theory in the critical aim of the study and to interpret empirical findings. The chosen approach for this study aims to push the research agenda that Guthold and colleagues (9) mention, concerning what drives gender inequalities, even further towards a more critical perspective. Here, we do this by investigating how the volume of sedentary time and physical activity in higher intensities performed among adolescent relate to their parents' activity and having siblings, both between and within gender categories. This will be done through both the use of (more common) linear dyadic models, as well as explorative nonlinear multivariate models.

## 3 Aim

The intention of this study is to scrutinize how gender and family structures relate to time spent sedentary and physically active when explored from a *doing gender* perspective. This will be done through analyzing the relationships between adolescents' leisure physical activity and time spent sedentary with their parents' activity and having siblings, between and within gender categories.

### 3.1 Research questions

1. What are the relationships between gender categories among parents' and adolescents' moderate-to-vigorous physical activity (MVPA) and time spent sedentary (SED) during leisure time on weekdays and weekends?
2. What are the relationships between gender categories among having siblings and adolescents' MVPA and SED during leisure time on weekdays and weekends?
3. How do the relationships between parents' and adolescents' MVPA and SED appear when stratified by parents' educational level? What are the inter- and intra-categorical structural relationships between adolescents' behaviors and their parents, in relation to educational level and gender?

## 4 Methods and materials

The current study is nested in a larger cross-sectional study (33). Adolescents together with their parents were invited to participate mainly through mail and e-mail, sent out both through the schools and via home addresses. All schools were situated within a radius of two-three hours driving from the larger urban area of Stockholm, Sweden. The sample was based on a variation of municipality type, based on the Swedish municipality classification 2017 with three groups (34). The first two groups; large cities and municipalities near large cities and medium-sized towns and municipalities near medium-sized towns, were categorized as urban areas. The third group including smaller towns/urban areas and rural municipalities was categorized as rural areas. The sample was also based on a variation of areas with low, middle, and high socioeconomic characteristics. In total, 1,556 adolescents from grade 7 (aged 13–14) and their parents were invited to participate in the study. After 351 who declined, 63 who were absent, and 3 who did not complete their consent form, a final sample of 1,139 (73%) adolescents and their parents, divided over 34 schools, were included in the study. All participating adolescents were compensated for participating with a gift card; however, parents were not.

The study was carried out in compliance with the Declaration of Helsinki and the protocol was approved by the Swedish Ethical Review Authority in Stockholm, Sweden (Dnr: 2019-03579). Before participating in the study, all adolescents with their parents gave their informed consent.

### 4.1 Data collection

Data was collected by a team of trained researchers and assistants from the Swedish School of Sport of Health Sciences (GIH) during the fall of 2019. Adolescent MVPA and SED were measured by accelerometry, and parental MVPA and SED were assessed through a questionnaire that was distributed via e-mail. Accelerometer monitors were distributed during the adolescents' visit at GIH, then collected by class teachers after seven days and sent back to GIH via pre-paid envelopes. All background characteristics for adolescents and their parents were assessed through self-reported questionnaires. However, parental educational level was retrieved from Statistics Sweden (SCB).

#### 4.1.1 Family and gender

We have chosen to include a broader understanding of the concept of family than just biological. The questionnaires to parents asked the question “What is your relationship to the participant in this study? The possible answers were “I am the mother or equivalent to the participant” and “I am the father or equivalent to the participant”. This way other forms of “mothering” and “fathering” guardians have been included. Similarly, the questions to participants regarding the numbers of sisters and brothers included half-siblings, step-siblings, and foster-siblings. The sibling-variables were dichotomized into having/not having sisters and brothers. The number of households lived in was assessed through asking the adolescents if “they lived in more than one household?”, providing them with two possible ways to answer; “yes” and “no”.

Adolescent participants have reported gender as either girl, boy, or other as a non-binary alternative. However, only one person chose the non-binary alternative, and therefore had to be excluded from the analyses.

#### 4.1.2 Adolescents physical activity and time spent sedentary

Data about physical activity and time spent sedentary were collected via triaxial accelerometers (model GT3X+, Actigraph, LCC, Pensacola, FL, USA). The participants received the accelerometers with written instructions and received an introduction by trained staff. Participants were instructed to always wear the accelerometer when awake, on their right hip, during the seven consecutive measuring days, except for activities in water (e.g., showering, swimming). A total of 2.3% of the adolescents reported aquatic sports as their activity, including swimming, diving and sailing.

The monitors collected activity data at a sampling rate of 30 Hertz and were down-sampled into 5 s time intervals for analysis, using ActiLife version 6.13.3. Non-wear time was removed, which was defined as 60 consecutive minutes or more of zero counts with zero-minute spike tolerance using triaxial data. A day was considered valid when 500 min of data or more remained after removing non-wear time. For the assessment to be valid, at least two days of measurement during leisure time weekdays as well as one day during the weekend were required. Uniaxial counts from the vertical axis of the accelerometer data were categorized into minutes spent in SED (0–100 counts/minute) and MVPA (≥2,296 counts per minute) (35). Additionally, the wear time in minutes was calculated and averaged across the included days.

Filters were created with two time domains, based on class-schedules and reported awake/asleep time from questionnaires where a) the time between the end of the school day and asleep time was defined as “leisure weekdays” and b) weekend time between time awake and asleep time was defined as “weekends”. Four missing class schedules were replaced with a schedule from another class in the same grade from the same school. The first day of registration was not used in the analysis to limit measurement bias. When dividing adolescents' time spent in MVPA and SED in high and low categories we split the variables into quartiles, where the two lowest represent “low” and the two highest represent “high”.

#### 4.1.3 Parents physical activity and time spent sedentary

**Figure 1:**
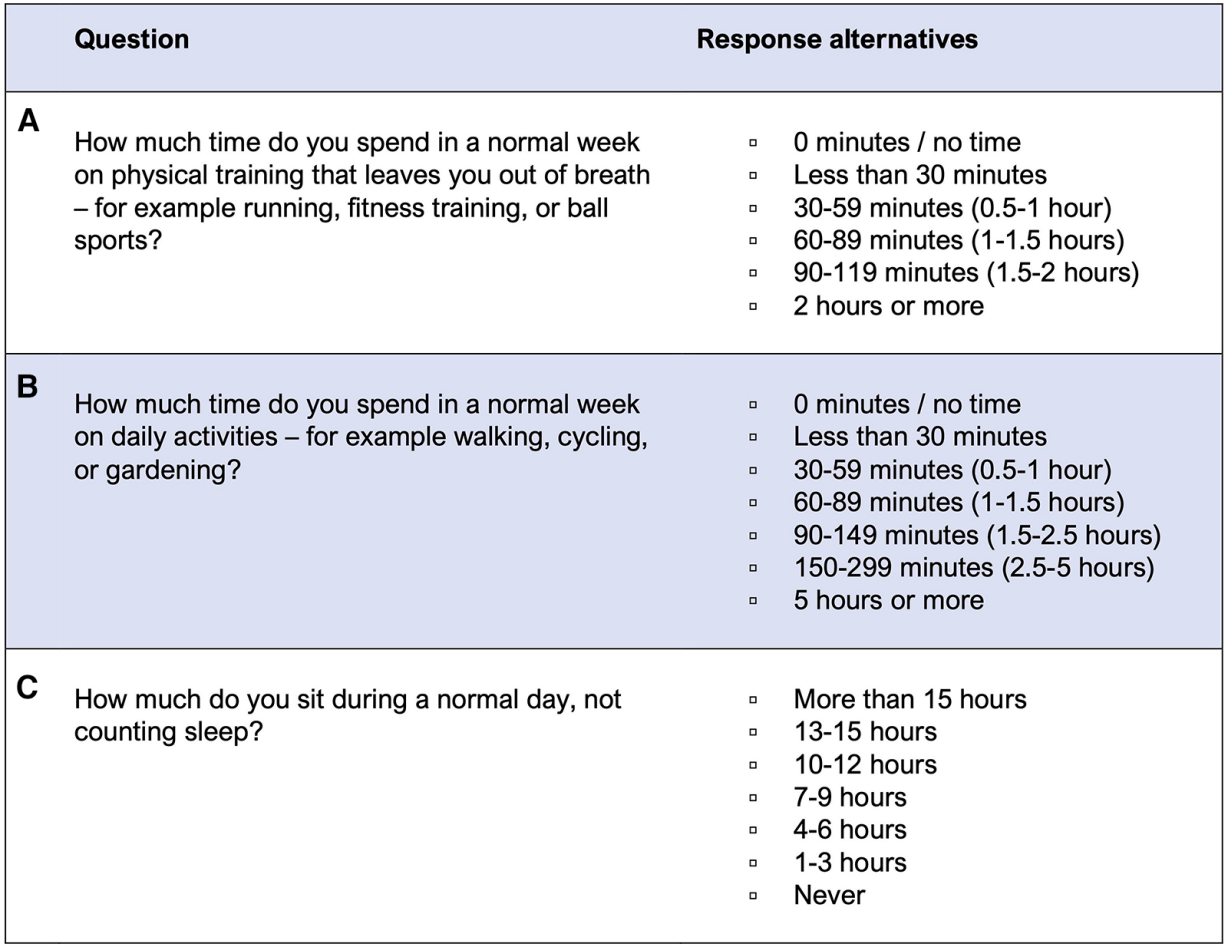
Questionnaire items for parents' physical activity.

Questions concerning parental MVPA and SED were self-reported. The questionnaire included three items, originally designed by the Swedish National Board of Health and Welfare (the NBHW) (36). The questions asked are displayed in Figure 1.

The physical activity answers (question A and B) were handled as a scale ranging from 3 to 19, as validated (concurrent and predictive validity) against accelerometry among Swedish adults (36). This scale was calculated from the number of minutes from question A*2 plus the number of minutes from question B, and is considered as parental MVPA. Every step up on the scale applied represents approximately 30 min MVPA per week added. For assessing parents reaching the recommendation of 150 min per week of MVPA, we dichotomized the variable into “not reaching” (scale 3–9) and “reaching” (scale 10–19).

The answers for time spent sedentary (question C) were handled in accordance to how they have been validated (concurrent and convergent validity) against accelerometry among Swedish adults (37). Therefore, the two categories “More than 15 h” and “13–15 h” were collapsed into one category, as well for “1–3 h” and “Never”. We additionally created a second dichotomized variable for assessing parents as “sedentary” (>10 h) or “not sedentary” (<10 h).

#### 4.1.4 Parental educational level

Educational level among parents were retrieved from publicly available data from Statistics Sweden (SCB) and used as an indicator for socioeconomic status (SES)/social marker. The variable was further dichotomized into low education (≤12 years) and high education (>12 years).

#### 4.1.5 Foreign background

Country of birth and foreign background were self-reported by adolescent participants, where they were asked to select, both for themselves and their parents, between four alternatives: born in Sweden, in other Nordic countries, in Europe, or outside Europe. The two categories Sweden and other Nordic countries were collapsed into one. Foreign background was further treated as two categories; Swedish-born (with at least one parent born in Sweden) or born outside Sweden (or both parents born outside Sweden).

#### 4.1.6 Statistical analysis

Descriptive statistics are reported in proportions for categorical variables, while continuous variables are reported in means with standard deviations. Linear regression models were used to assess associations between parents' MVPA and SED, having siblings, and adolescents' MVPA and SED, separately for boys and girls. All regression analyses were checked for normality and homoscedasticity and adjusted for accelerometer wear-time. The level of statistical significance was set conservatively to *p* < 0.01. Further, the linear models looking at maternal and paternal time in MVPA and SED as exposures were stratified by educational level. If any significant results were found in the stratified models interaction effects were tested by adding an interaction term to the unstratified models.

The variables were additionally explored using a non-linear multivariate correspondence analysis (38). The purpose was to visualize an inter- and intra-categorical map over the adolescents and their parents' MVPA behaviors and their mutual relationships, in connection to educational level, to further assess how MVPA is expressed in relation to gender in this specific context. A contingency Table (A) was used for this analysis (see Supplementary Material 1).

**Figure 2:**
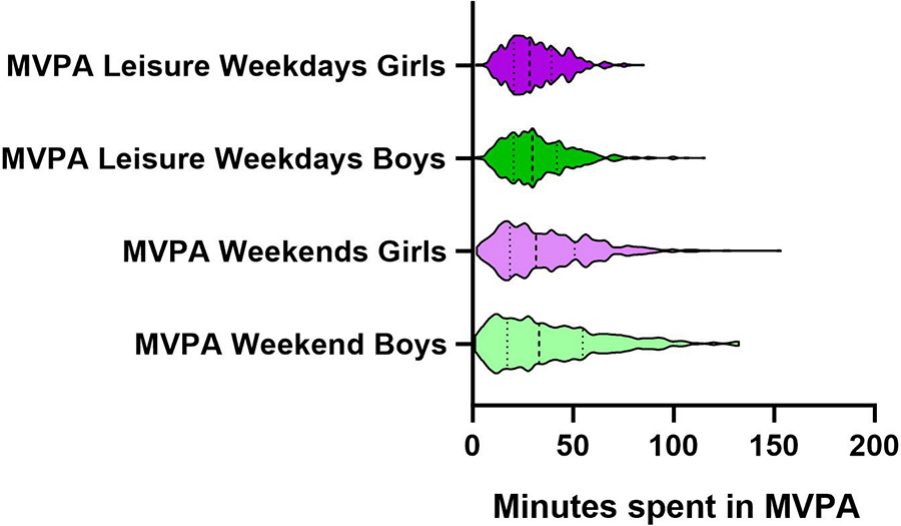
Minutes spent in leisure moderate-to-vigorous physical activity among adolescents.

**Figure 3:**
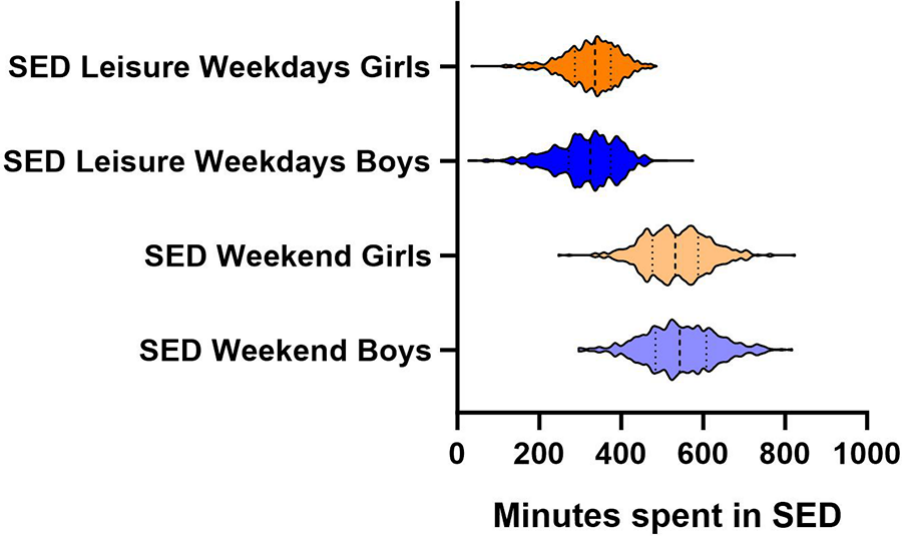
Minutes spent sedentary during leisure time among adolescents.

**Figure 4:**
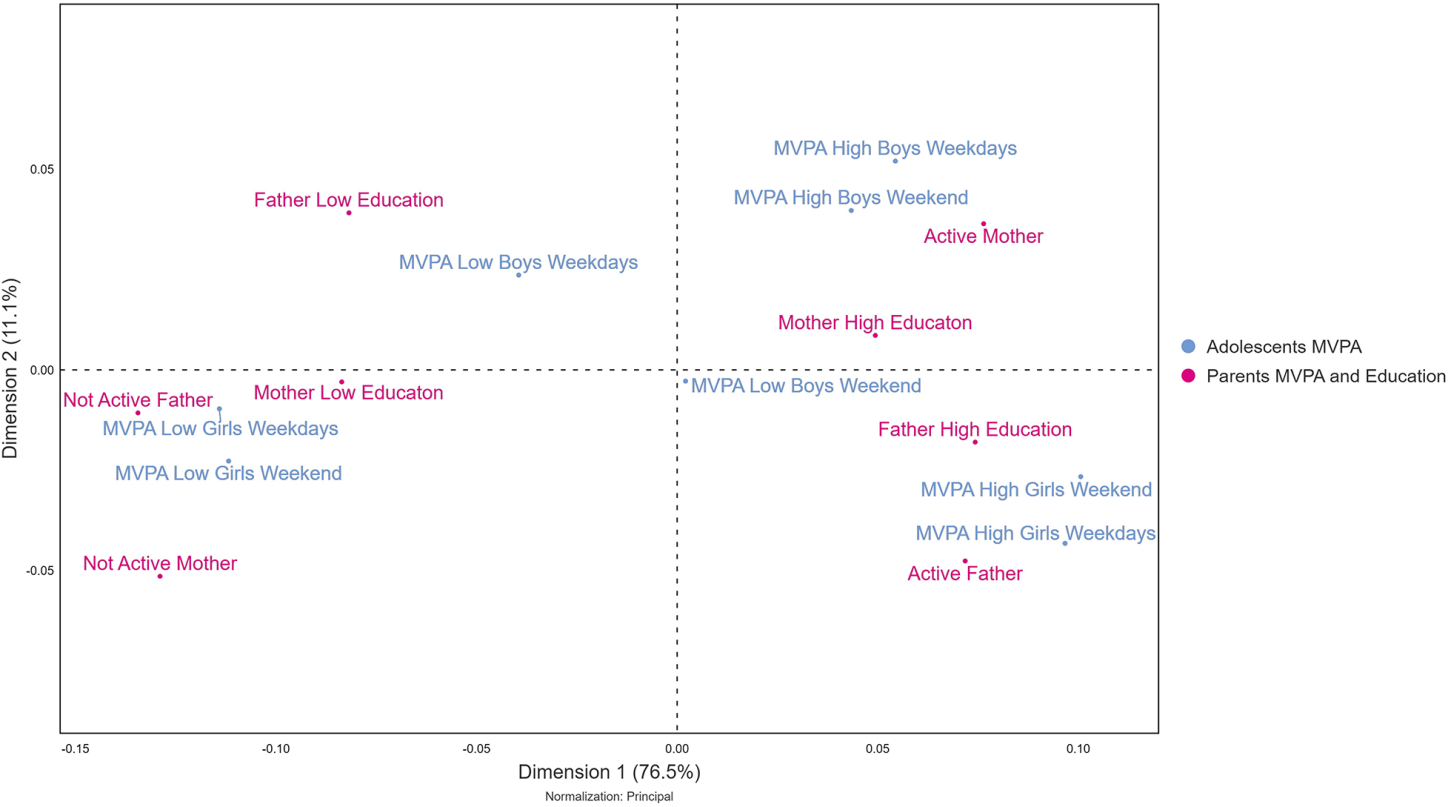
Correspondence analysis.

The IBM SPSS Statistics for Windows software, v. 27, and STATA/SE v.17.0 was used for the statistical analyses. For the visualizations, GraphPad software was used for the violin plots in Figures 2, 3, and Display software was used for correspondence analysis in Figure 4.

### 4.2 Sample characteristics

The total sample included 1,139 adolescent participants, with an even distribution between girls (49.0%) and boys (51.0%). The mean (SD) age was 13.4 (0.4) years. The proportion of adolescents in the sample with foreign background was 28.4%, and living in two households (instead of one) was 15.9%. The proportion of adolescents that have sisters was 63.0% and brothers 67.8%. Of the whole sample, 1,054 (92.6%) of the participants had valid accelerometer registrations for weekdays and 895 (78.7%) for weekends. The distribution of MVPA and SED among adolescents is shown in Figure 2 (MVPA) and Figure 3 (SED), displaying the variation within and between gender categories showing the median and quartiles of the sample. The mean (SD) MVPA on weekdays was 31.61 (15.0) minutes per day and on weekends 37.9 (25.3) minutes per day, and the mean (SD) SED on weekdays was 324.8 (69.3) minutes per day and 539.5 (84.7) minutes per day on weekends. A statistically significant difference between girls and boys was found for SED, however, no other statistical significance was found between girls and boys. All results are presented further in Table 1.

**Table 1:** Descriptive statistics for adolescents.

	All *n* (%)	Girls *n* (%)	Boys *n* (%)	*p*
Number of adolescents	1,139 (100)	580 (51.0)	558 (49.0)	
Age (mean ± SD)	13.4 ± 0.4	13.4 ± 0.3	13.4 ± 0.4	0.147
Foreign background				
Swedish born, and at least one Swedish born parent	800 (71.6)	414 (72.5)	386 (70.8)	0.534
Born outside Sweden, or Swedish born and both parents born outside of Sweden	317 (28.4)	157 (27.5)	159 (29.2)	
Number of households				
Living in one household	953 (84.1)	494 (85.3)	458 (82.8)	0.251
Living in two households	180 (15.9)	85 (14.7)	95 (17.2)	
Having sisters				
No sisters	378 (37.0)	203 (38.8)	175 (35.1)	0.224
Have sisters	644 (63.0)	320 (61.2)	323 (64.9)	
Having brothers				
No brothers	327 (32.2)	173 (32.5)	154 (32.0)	0.847
Have brothers	687 (67.8)	359 (67.5)	328 (68.0)	
MVPA (mean ± SD)				
Leisure weekdays	31.6 (15.0)	30.9 (13.8)	32.3 (16.1)	0.145
Weekends	37.9 (25.3)	36.8 (23.3)	39.1 (27.4)	0.156
SED (mean ± SD)				
Leisure weekdays	324.8 (69.3)	330.0 (63.5)	319.2 (74.7)	0.011
Weekends	539.5 (84.7)	533.8 (80.0)	546.1 (89.6)	0.029

The total sample of parents included 815 mothers and 572 fathers. Adolescents who had data from both their mother and father were 438, representing 38.5% of the sample. The mean (SD) age for mothers was 44.4 (4.8) years and fathers 47.6 (5.5) years, and the proportion of having 12 years education or more among mothers were 55.2% and 47.3% among fathers. The proportion of parents reaching the recommendation of >150 min of MVPA per week was 67.2% of mothers and 73.2% of fathers, and SED more than 10 h per day among mothers was 56.8% and fathers 48.4%. All descriptive data for parents is presented in Table 2.

**Table 2:** Descriptive statistics for parents.

	Mothers *n* (%)	Fathers *n* (%)
Number of parents	815 (58.8)	572 (41.2)
Age (mean ± SD)	44.4 (4.8)	47.6 (5.5)
Highest education		
Up to 12 years	492 (44.8)	563 (52.7)
More than 12 years	607 (55.2)	505 (47.3)
Reaching physical activity (MVPA) recommendation 150+ minute/week		
Yes	542 (67.2)	415 (73.2)
No	264 (32.8)	152 (26.8)
SED		
Less than 3 h per day	25 (3.1)	23 (4.0)
4–6 h per day	76 (9.4)	83 (14.6)
7–9 h per day	249 (30.7)	188 (33.0)
10–12 h per day	267 (33.0)	192 (33.7)
More than 13 h per day	193 (23.8)	84 (14.7)

## 5 Results

### 5.1 Mothers' and fathers' MVPA and SED in relation to adolescents'

The results from the linear regression models showed positive relationships between mothers' MVPA and girls MVPA during weekdays (*ß* = 0.54, 99% CI: 0.15, 0.94, *p *= 0.000) and weekends (*ß* = 0.78, 99% CI: 0.09, 1.46, *p *= 0.004). Similar positive associations were found between fathers’ MVPA and girls MVPA during weekdays (ß = 0.49, 99% CI: 0.00, 0986, *p *= 0.010). The results imply that every increase of approx. 30 min among mothers self-reported MVPA per week is related to an increase of 0.54 min per day in accelerometer measured MVPA among girls during leisure time, and also an increase of 0.78 min per day among girls MVPA on weekends. Likewise, every increase of approx. 30 min per week among fathers' MVPA is associated with an increase of 0.49 min per day in MVPA among girls on weekdays. No associations were found between mothers' or fathers' SED and girls' or boys' SED. All results are displayed in Table 3.

**Table 3:** Associations between parents’ MVPA/SED and adolescents’ leisure MVPA/SED.

	Girls		Boys	
	ß (99% CI)	*R* ^2^	ß (99% CI)	*R* ^2^
Leisure MVPA weekdays				
Mothers’ MVPA	**0.54** **(0.15, 0.94)**	**0** **.** **06**	0.41 (−0.05, 0.87)	0.06
Fathers’ MVPA	**0.49** **(0.00, 0.98)**	**0** **.** **03**	0.03 (−0.52, 0.58)	0.04
MVPA weekends				
Mothers’ MVPA	**0.78** **(0.09, 1.46)**	**0** **.** **06**	0.53 (−0.34, 1.41)	0.04
Fathers’ MVPA	−0.08 (−0.90, 0.74)	0.01	−0.02 (−1.04, 1.00)	0.01
Leisure SED weekdays				
Mothers’ SED				
Less than 3 h per day	REF	0.80	REF	0.82
4–6 h per day	−2.68 (−12.07, 6.70)		−1.19 (−13.00, 10.62)	
7–9 h per day	−3.13 (−12.77, 6.51)		−0.98 (−12.57, 10.60)	
10–12 h per day	6.86 (−7.92, 21.63)		−4.56 (−19,97, 10.84)	
More than 13 h per day	0.15 (−20.23, 20.52)		−19.90 (−46.45, 0.88)	
Fathers’ SED				
Less than 3 h per day	REF	0.78	REF	0.82
4–6 h per day	3.38 (−9.45, 16.20)		4.03 (−13.06, 21.10)	
7–9 h per day	0.90 (−11.98, 13.77)		3.34 (−13.62, 20.31)	
10–12 h per day	2.28 (−12.99, 17.54)		3.28 (−16.12, 22.67)	
More than 13 h per day	14.70 (−8.20, 37.60)		−11.35 (−44.42, 21.73)	
SED weekends				
Mothers’ SED				
Less than 3 h per day	REF	0.62	REF	0.56
4–6 h per day	−2.50 (−19.26, 14.26)		1.90 (−23.23, 27.04)	
7–9 h per day	−2.08 (−19.32, 15.15)		1.24 (−23.73, 26.20)	
10–12 h per day	5.53 (−20.69, 31.74)		−11.85 (−45.20, 21.49)	
More than 13 h per day	8.62 (−27.55, 44.79)		−37.39 (−90.70, 15.91)	
Fathers’ SED				
Less than 3 h per day	REF	0.63	REF	0.62
4–6 h per day	0.39 (−22.02, 22.81)		8.86 (−27.00, 44.71)	
7–9 h per day	−9.88 (−32.41, 12.66)		23.23 (−12.07, 58.53)	
10–12 h per day	−0.01 (−26.92, 26.90)		21.31 (−18.93, 61.54)	
More than 13 h per day	19.52 (−20.77, 59.81)		7.89 (−59.90, 75.68)	

Models are adjusted for accelerometer wear time. Results in bold are statistically significant *α* < 0.01.

### 5.2 Having sisters or brothers and adolescents' MVPA and SED

The results showed negative associations between having brothers and boys' SED during weekends (*ß* = −18.33, 99% CI: −35.24, −1.42, *p *= 0.005). No other associations were found significant between having brothers or sisters and boys or girls MVPA and SED. The results indicate that having brothers for boys was related to −18 min less SED during weekends. All results are displayed in Table 4.

**Table 4:** Associations between adolescents having siblings and their time spent in MVPA and SED.

	Girls		Boys	
	ß (99% CI)	*R* ^2^	ß (99% CI)	*R* ^2^
Leisure MVPA on weekdays				
Having sisters	−0.51 (−3.76, 2.74)	0.03	2.71 (−1.20, 6.61)	0.06
Having brothers	0.47 (−2.88, 3.81)	0.04	−0.50 (−4.55, 3.55)	0.05
MVPA on weekends				
Having sisters	1.19 (−4.62, 7.01)	0.02	6.51 (−1.26, 14.27)	0.03
Having brothers	−0.37 (−6.29, 5.56)	0.04	3.24 (−4.44, 10.91)	0.02
Leisure SED weekdays				
Having sisters	0.58 (−5.78, 6.93)	0.83	−4.58 (−12.42, 3.26)	0.83
Having brothers	−2.14 (−8.70, 4.43)	0.83	−6.76 (−14.76, 1.24)	0.84
SED weekends				
Having sisters	−2.53 (−14.72, 9.67)	0.63	−5.87 (−22.83, 11.10)	0.56
Having brothers	−5.69 (−18.18, 6.81)	0.63	**−18.33 (−35.24, −1.42)**	**0** **.** **56**

Models are adjusted for accelerometer wear time. Results in bold are statistically significant *α* < 0.01.

### 5.3 Associations in relation to educational level

We further explored how the relationship between parents and adolescents MVPA appear when stratified by parents' educational level. Statistically significant positive results were only found between highly educated mothers MVPA and girls MVPA during weekdays (*ß* = 0.71, 99% CI: 0.16, 1.13, *p *= 0.001) and weekends (*ß* = 1.20, 99% CI: 0.24, 2.16, *p *= 0.001). On the contrary, positive results were found between low-educated mothers MVPA and boys MVPA on weekdays (*ß* = 0.93, 99% CI: 0.18, 1.70, *p *= 0.002). Significant relations were also found between highly-educated fathers MVPA and girls MVPA on weekdays (*ß* = 0.74, 99% CI: 0.17, 1.46, *p *= 0.008). When we ran interaction analyses, it did not show any significant results for any of these associations, with educational level and maternal MVPA for girls MVPA on weekdays (*p *= 0.087) and weekends (*p *= 0.044), and low-educated mothers MVPA and boys MVPA on weekdays (*p *= 0.022) There were also no significant interaction between highly-educated fathers MVPA and girls MVPA on weekdays (*p *= 0.186). No other significant results were found between parents MVPA and adolescents MVPA. After stratified by education the results for SED were not significant over all categories (see Table 2, Supplementary Material 2).

### 5.4 Gendered inter- and intra-relational dispositions

Our explorative non-linear correspondence analysis is displayed in Figure 4. It displays the relations between parents and their adolescents' physically active (intensity) behaviors, also taking educational level into account. Here, dimensions 1 and 2 together explain 87.6% of the variance. The visualisation is based on a contingency table (see Table 1, Supplementary Material 1), where the plot-labels with similar residuals are pictured close together. Figure 4 shows that girls with high and low levels of MVPA seem to be distinct from each other, as well as both being distinct in relation to the origin. Boys with high levels of MVPA are more distinct from the origin the boys with low levels of MVPA, however, these two are not as distinct from each other as shown for girls. Active mothers and fathers are seemingly related to girls having higher levels of MVPA, both on weekdays and weekends. Active parents with higher education give the impression of being closer connected to girls and boys with higher levels of MVPA. Girls with lower levels of MVPA, both on weekdays and weekends, are related to mothers with low education as well as both less active mothers and fathers. The educational level of parents appears to relate somewhat to boys' low levels of MVPA on weekdays.

## 6 Discussion

This cross-sectional study's aim was to explore gendered structures among parents and siblings in relationship to adolescents' time spent being sedentary and physically active, using West and Zimmerman (29) theoretical lens of *doing gender*.

The results showed statistically significant relationships between mothers' MVPA and girls MVPA on both weekdays and weekends. However, fathers' MVPA were only significantly related to girls' MVPA on weekdays. This suggests that there are gendered patterns among parents and their adolescent children in relationship to MVPA, however not for SED where no significant associations were found. Expressed in terms of “doing gender”, this means that while SED never signifies doing gender, at least not in this sample, MVPA sometimes does. When stratified for educational level, statistically significant results suggest that the patterns between parents MVPA and adolescents are somewhat gendered in relation to other social dispositions such as education. The current study's results for having siblings only showed significant relationships between having brothers and less SED for boys during weekends. When exploring these relationships from a non-linear and intra-categorical model using correspondence analysis the plot was interpreted with support from a contingency table with adolescents MVPA variables in rows and parental variables in columns, showing how they relate to each other. The interpretation displays how levels of MVPA and educational level of parents intersects slightly more often with girls' levels of MVPA, where lower levels among parents relate to lower levels among girls as well as the opposite. Girls with high and low levels of MVPA seem to be more distinct from each other, in relation to boys with high and low levels of MVPA.

The positive relationships on parents MVPA reported are somewhat in line with earlier research (19). Though, Petersen and colleagues (19) “found little evidence across studies to suggest a gender-differentiated size of resemblance in physical activity between parents and their children”, though, the studies included in their review were few and most of them were of poor quality. Additionally, most studies reported only mother-child, father-child or parent-daughter/son relationships. Edwardson and Gorely (39) showed that mothers' and fathers’ physical activity was associated with adolescent MVPA, yet the relationship between gender combinations seems rather indeterminate. Our results are both similar and conflicting with earlier research from Finland (25). The results are conflicting in the way that they showed that fathers' activity strongly related to their adolescents' activity levels, and similarly in the way that mothers' high to moderate activity levels increased the likelihood of their daughters being physically during youth. This signifies that what is doing gender in one context/country, is not necessarily the same in another one. A recent Swedish study evaluated longitudinal patterns and correlations of physical activity between parents and their children, where results showed a positive significant correlation over time between child physical activity and maternal physical activity from three to six years of age (40). These results are similar to the current study's results. Earlier research reports that the number of siblings influenced sedentary behavior, however, no gender differences were reported (41). This contributes to limited comparisons between the studies and calls for further investigations of influencing aspects of gender. Other factors (e.g., cognitive and genetic) than gendered parentage and siblingship might also influence the reported inequalities in levels of physical activity and sedentary behaviors between girls and boys. Further, how gender is performed and institutionalized in differing cultures and contexts are aspects to consider when comparing future study results and a potential factor to explore, as well as changes over time.

From a socialization perspective, there are other aspects of family and physical activity than activity levels that might have an influence. For example, Strandbu and colleagues (42) investigated the importance of “family sport culture” during teenage years among Norwegian youths, showing a clear positive relationship between family sport culture and participation in club-organized sports. Here, parents' training habits, together with the perceived importance of sport and whether parents would like their children to participate in sports was aggregated into one measure. The relationship was equally strong in all age groups, except for that the relationship was slightly weakened with age among girls (42). This highlights the importance of other aspects of parenting and family socialization in relation to sport and physical activity. Our exploratory results within a Swedish context imply that “mothering” and “fathering” that involves lower and higher levels of MVPA is related to girls' lower or higher level of engagement in MVPA. This could mean that boys' engagement in MVPA is not in the same “need” of the relationship to parents and their MVPA to the same extent as girls, and that the drivers of being physically active potentially lies somewhere else. Boys physical activity behaviors and having access to the possibilities of being active (with high intensities) is more in line with traditional gender roles, where girls on the other hand “must negotiate the transgression of traditional gender norms to participate in physical activity” (43).

Gender theory concerns bodies, where gender practices can be considered as reflexive processes of social embodiment which are mirrored in how societies handles social activities such as motherhood and fatherhood as well as sport and physical activity (13). Bodies can also be recognized as being involved in social class processes. When we further assessed how educational level intersects with MVPA and gender, the exploratory results imply that the “doing” of gender in relation to MVPA practices are to some extent carried out differently depending on educational level. However, these very explorative results should be interpreted with caution. As an example, Clark (44) emphasizes the implications that postfeminist and healthism discourses have on women's understandings of their bodies and participation in sport and physical activity, and how this represents a cultural shift toward the understanding that bodies and their health is an increasingly personal and individualized responsibility. This further creates distinctions where girls' and women's participation in physical activity is viewed as “successful” through a logic of self-improvement and gender empowerment, where physically active bodies serve as markers of distinction and accomplishment within competitive hierarchies (44). However, the interesting aspect here is that “femininities” involving both lower and higher levels of MVPA seem to intersect with parents with activity behaviors, additionally in relation to level of education. As Clark (44) discusses “successful girlhood”, where physical activity becomes embodied as a social marker. Altogether, our results should be interpreted sensibly to be further explored in future research, exploring “successful femininities” across ages in relation to physical activity behaviors in higher intensities.

To our knowledge, there are not many quantitative attempts to assess the aspect of *doing gender* in relationship to physical activity and sedentary behavior among youth. Our overall analysis is limited in its scope since it addresses dyadic data and binary gender categories, as well as an explorative non-linear way of looking at how different variables intersect within groups. However, it offers a perspective for how the relationship between parents, siblings and adolescents' physical activity and sedentary behavior can be interpreted from a non-essentialist approach, both using standard methods and alternative ones. This perspective contributes to a further understanding of the drivers of family and gender in the context of physical activity behaviors, particularly in relation to volume and levels of intensity and what they can mean. With that in mind, our results do not mean that we should target a specific gender category for physical activity interventions, or for that matter, that e.g., mothers and brothers bear inherently essential characteristics of physical activity role-modelling. Instead, our results suggest that the “doing” of physical activity (here, especially MVPA) as a social practice is connected to (re)productions of gender e.g., through “mothering”, “fathering”, “daughtering”, and “brothering”. Our results imply that there are gendered patterns to further consider, relating to adolescents' physically active life between and within gender categories, at least in a Swedish context. We hope that the outcomes of this study support pushing the research agenda mentioned by Guthold and colleagues (9) on health inequalities relating to physical activity and young people's lives even further. A few suggestions for this push forward include (1) adding more extensive assessments of family variables and gender combinations in quantitative data analyses, that make use of (2) non-essentialist perspectives and gender-theory, and (3) assesses the variations of how physical activity is expressed, together with (4) the addition of qualitative and/or mixed-methods that can further explore the relationship between gender, family and physical activity to deepen our understanding of these phenomena. It could also involve an intersectional perspective, where “doing gender” is systematically analyzed in relation to “doing class” and “doing ethnicity”, for example.

From our perspective this study contributes in several ways, nonetheless, as earlier touched upon, it also has its limitations. A strength of the current study is the large sample size, as well as the novelty of a non-essentialist gender-theory informed quantitative approach. Another strength of the study is that it includes both data on mothers, fathers and sibling status. Accelerometry was used to assess time spent in physical activity and sedentary, which is a valid and reliable method. Conversely, in relationship to the chosen “doing gender” theory for this study accelerometry is limited in its scope where variations in the expression of physical activities is not considered for. Therefore, the scope was limited to how physical activity in relation to higher intensities is potentially gendered, specifically within adolescents-parent dyadic data. To further integrate the theoretical approach with the analysis, we performed an explorative non-linear multivariate analysis where intra- and inter-categorical data was assessed. This way, our scope also included aspects of intensity in physical activity between and within gender categories. Regarding the limitations of the correspondence analysis, e.g., not displaying any information concerning statistical significance, the visualization and our interpretations are explorative and should be further interpreted with that notion in mind. Although, the inferences made has been cross-checked in relation to the original data and our initial linear analyses.

With cross-sectional study designs there are always the limitation of data being tied to only one timepoint. From our theoretical approach, this limits the analysis concerning the temporal aspect of “doing gender” and how physical activity is performed in relation to gender over time. In our sample, there was a higher response rate among mothers than fathers, and the participants who chose to be part of the study could potentially also be more positive towards physical activity in relation to those who chose not to participate. However, the results showed that participants were rather representative in regard to background characteristics which increases the study's transferability, at least within a Swedish context.

Earlier research suggests several ways parents and family can influence and support physical activity practices; e.g., parents' individual level of physical activity or co-activity with their child (modeling), parents level of promoting or persuading physical activity for their child (encouragement and emotional support), and transport to/from physical activities or facilitating equipment (instrumental), household practices and environment, and family beliefs, attitudes and knowledge (16, 45). In the current study, only the volume of MVPA among parents and adolescents was reported, without knowing if these activities co-occurred at the same time and space. Also, only the existence of brothers or sisters was reported. Future research should aim to gather more extensive data on adolescents' physical activity behaviors, and parents and siblings (e.g., age and physical activity behaviors), as well as their sociocultural and economic context. These are all aspects that can be further explored in future research on this topic from a gender-theory perspective.

## 7 Conclusion

Our results imply that Swedish parents’ and adolescent girls' physical activity in higher intensities is to some extent related. However, time spent sedentary does not seem to show any patterns of being performed according to binary ideas of gender among adolescents and their parents. Further, our exploratory analyses suggest that these results somewhat intersect with parents’ educational level between and within groups. Especially, we highlight how physical activity in higher intensities as part of “doing gender” (e.g., successful femininities) are critical topics to be further studied. The present study also suggests limited gendered patterns in the “doing” of siblinghood for time spent sedentary, however, for boys only on weekends.

We hope that this explorative, yet important, contribution to the understanding of gender norms as constraints and enablers for adolescents' participation in physical activity can spur public health and physical activity research to further apply a contemporary gender theory approach. Lastly, our ambition is to postulate potential entrances to future areas of investigation as well as to expand the research agenda connected to what drives gender inequalities in physical activity practices. One way to further explore a *doing gender* perspective in future physical activity research could be to assess the intensity and quantity of physical activity through accelerometry combined with assessing variations in the expression of physical activities. In addition, gender theory could be incorporated in more ways, for example as part of the original study design (a mentioned limitation within the current study). This could advance the investigations of both inter- and intra-categorical aspects of gendered patterns and norms connected to physical activity.

## Data Availability

The dataset for this study is not made public due to confidentiality towards the participants. Anonymous data for future analyses can be provided upon request from the principal investigator. Requests to access the datasets should be directed to GN, gisela.nyberg@gih.se.
