# Supplementary material for: Gendered relations? Associations between Swedish parents, siblings, and adolescents' time spent sedentary and physically active
Source: Front Sports Act Living. 2024 Feb 22;6:1236848. doi: 10.3389/fspor.2024.1236848 (PMC10918000; doi:10.3389/fspor.2024.1236848)
Supplement: Supplementary file 1 [file Table1.docx]

**Supplementary Material 1**

**Table 1. Contingency table in counts used for the correspondence analysis.**

|  |  | **Mothers reaching MVPA recommendation or not** | | **Fathers reaching MVPA recommendation or not** | | **Mothers education being more or less than 12 years** | | **Fathers education being more or less than 12 years** | |
| --- | --- | --- | --- | --- | --- | --- | --- | --- | --- |
|  |  | Not active mother | Active mother | Not active father | Active father | Mother low educaton | Mother high educaton | Father low education | Father high education |
| **Girls MVPA weekdays** | Girls low MVPA weekdays | 79 | 123 | 49 | 98 | 137 | 140 | 149 | 117 |
|  | Girls high MVPA weekdays | 57 | 127 | 27 | 114 | 99 | 146 | 110 | 129 |
| **Girls MVPA weekend** | Girls low MVPA weekend | 72 | 107 | 41 | 92 | 122 | 124 | 134 | 104 |
|  | Girls high MVPA weekend | 50 | 126 | 32 | 103 | 92 | 138 | 99 | 121 |
| **Boys MVPA weekdays** | Boys low MVPA weekdays | 70 | 123 | 35 | 95 | 110 | 152 | 146 | 113 |
|  | Boys high MVPA weekdays | 43 | 125 | 29 | 86 | 98 | 128 | 113 | 109 |
| **Boys MVPA weekend** | Boys low MVPA weekend | 55 | 99 | 25 | 75 | 86 | 119 | 103 | 99 |
|  | Boys high MVPA weekend | 43 | 111 | 27 | 78 | 83 | 114 | 101 | 94 |
