# Supplementary material for: Gendered relations? Associations between Swedish parents, siblings, and adolescents' time spent sedentary and physically active
Source: Front Sports Act Living. 2024 Feb 22;6:1236848. doi: 10.3389/fspor.2024.1236848 (PMC10918000; doi:10.3389/fspor.2024.1236848)
Supplement: Supplementary file 2 [file Table2.docx]

**Supplementary Material 2**

**Table 2. Associations between parents’ MVPA / SED and adolescents’ leisure MVPA / SED, stratified by parental educational level**

|  | **Leisure MVPA weekdays** | | | |
| --- | --- | --- | --- | --- |
|  | **Girls** | | **Boys** | |
|  | ß (99% CI) | R² | ß (99% CI) | R² |
| Mothers’ MVPA |  |  |  |  |
| < 12 years education  > 12 years education | 0.18 (-0.42, 0.78)  **0.71 (0.16, 1.13)** | 0.03  **0.08** | **0.93 (0.18, 1.70)**  0.11 (-0.48, 0.71) | **0.13**  0.04 |
| Fathers’ MVPA |  |  |  |  |
| < 12 years education  > 12 years education | 0.21 (-0.52, 0.94)  **0.74 (0.17, 1.46)** | 0.01  **0.05** | -0.00 (-0.86, 0.85)  0.09 (-0.66, 0.85) | 0.04  0.05 |
|  | **MVPA weekends** | | | |
|  | **Girls** | | **Boys** | |
|  | ß (99% CI) | R² | ß (99% CI) | R² |
| Mothers’ MVPA |  |  |  |  |
| < 12 years education  > 12 years education | 0.07 (-0.97, 1.11)  **1.20 (0.24, 2.16)** | 0.04  **0.07** | 0.33 (-1.07, 1.73)  0.56 (-0.61, 1.72) | 0.06  0.03 |
| Fathers’ MVPA |  |  |  |  |
| < 12 years education  > 12 years education | -0.46 (-1.66, 0.74)  0.12 (-1.08, 1.32) | 0.03  0.00 | -0.27 (-1.91, 1.38)  0.54 (-0.79, 1.86) | 0.02  0.05 |
|  | **Leisure SED weekdays** | | | |
|  | **Girls** | | **Boys** | |
|  | ß (99% CI) | R² | ß (99% CI) | R² |
| Mothers’ SED  < 12 years education  < 3 hours per day  4-6 hours per day  7-9 hours per day  10-12 hours per day  > 13 hours per day  > 12 years education  < 3 hours per day  4-6 hours per day  7-9 hours per day  10-12 hours per day  > 13 hours per day | REF  3.46 (-10.28, 17.20)  -0.59 (-15.11, 13.93)  19.89 (-7.49, 47.28)  9.16 (-22.64, 40.96)  REF  -5.61 (-18.85, 7.64)  -3.88 (-17.21, 9.47)  3.52 (-15.04, 22.08)  -3.97 (-30.86, 22.89) | 0.85  0.76 | REF  6.23 (-13.48, 25.94)  18.78 (-0.60, 38.16)  5.21 (-19.66, 30.05)  -24.45 (-83.68, 34.78)  REF  -7.48 (-22.37, 7.42)  -12.64 (-27.22, 1.94)  -9.13 (-28.97, 10.71)  -22.49 (-52.29, 7.31) | 0.83  0.83 |
|  |  |  |  |  |
| Fathers’ SED  < 12 years education  < 3 hours per day  4-6 hours per day  7-9 hours per day  10-12 hours per day  > 13 hours per day  > 12 years education  < 3 hours per day  4-6 hours per day  7-9 hours per day  10-12 hours per day  > 13 hours per day | REF  5.86 (-12.07, 23.80)  6.91 (-12.17, 25.99)  9.15 (-14.97, 33.28)  19.06 (-27.71, 65.83)  REF  1.02 (-19.44, 21.48)  -3.07 (-23.08, 16.94)  -2.27 (-24.62, 20.08)  12.42 (-16.56, 41.39) | 0.77  0.80 | REF  2.89 (-19.13, 24.92)  -1.03 (-24.06, 22.00)  5.04 (-27.24, 37.33)  -12.81 (-63.39, 37.77)  REF  11.43 (-28.57, 51.44)  9.90 (-29.79, 49.59)  8.57 (-32.34, 49.49)  -4.57 (-59.03, 49.88) | 0.83  0.83 |
|  | **SED weekends** | | | |
|  | **Girls** | | **Boys** | |
|  | ß (99% CI) | R² | ß (99% CI) | R² |
| Mothers’ SED  < 12 years education  < 3 hours per day  4-6 hours per day  7-9 hours per day  10-12 hours per day  > 13 hours per day  > 12 years education  < 3 hours per day  4-6 hours per day  7-9 hours per day  10-12 hours per day  > 13 hours per day | REF  -2.62 (-27.28, 22.03)  7.19 (-19.21, 33.56)  22.83 (-32.95, 78.62)  7.42 (-48.90, 63.74)  REF  0.81 (-22.86, 24.49)  -4.41 (-28.23, 19.41)  3.73 (-28.31, 35.77)  12.81 (-35.33, 60.96) | 0.65  0.62 | REF  21.89 (-16.54, 60.32)  34.24 (-4.04, 72.53)  -17.82 (-69.44, 33.81)  -53.04 (-162.83, 56.76)  REF  -10.32 (-43.92, 23.28)  -16.74 (-49.92, 16.45)  -10.92 (-54.55, 32.71)  -37.32 (-99.98, 25.35) | 0.58  0.57 |
| Fathers’ SED  < 12 years education  < 3 hours per day  4-6 hours per day  7-9 hours per day  10-12 hours per day  > 13 hours per day  > 12 years education  < 3 hours per day  4-6 hours per day  7-9 hours per day  10-12 hours per day  > 13 hours per day | REF  1.40 (-30.67, 33.47)  -8.68 (-42.76, 25.39)  9.07 (-33.61, 51.75)  8.51 (-70.91, 87.94)  REF  -4.61 (-39.20, 29.98)  -14.48 (-48.27, 19.30)  -8.97 (-47.46, 29.51)  19.13 (-31.70, 69.95) | 0.59  0.66 | REF  12.52 (-36.36, 61.40)  12.37 (-35.71, 60.45)  8.22 (-61.18, 77,62)  -55.94 (-173.81, 61.93)  REF  -11.35 (-86.39, 63.68)  7.06 (-67.12, 81.23)  3.56 (-73.19, 80.30)  16.99 (-83.89, 117.86) | 0.59  0.62 |

Models are adjusted for accelerometer wear time. Results in bold are statistically significant α < 0.01.
